# From folklore to explore: integrating genomic and multiomics data for *Clinacanthus nutans* provides insights into the evolution and organ-specific therapeutic basis

**DOI:** 10.1093/hr/uhag037

**Published:** 2026-02-23

**Authors:** Chang An, Bingrui Wang, Denglin Li, Junzhang Li, Yongbin Lu, Yixin Yao, Yanxiang Lin, Lin Lu, Yan Cheng, Chongrong Ke, Zongshen Zhang, Ping Zheng, Yuan Qin

**Affiliations:** Fujian Provincial Key Laboratory of Haixia Plant Systems Biology, Center for Genomics, College of Life Science, Fujian Agriculture and Forestry University, Fuzhou 350002, China; College of Plant Science & Technology, College of Life Science and Technology, Huazhong Agricultural University, Wuhan 430070, China; Fujian Provincial Key Laboratory of Haixia Plant Systems Biology, Center for Genomics, College of Life Science, Fujian Agriculture and Forestry University, Fuzhou 350002, China; College of Plant Science & Technology, College of Life Science and Technology, Huazhong Agricultural University, Wuhan 430070, China; Yazhouwan National Laboratory, Sanya 572024, China; Guangxi Key Laboratory of Plant Conservation and Restoration Ecology in Karst Terrain, Guangxi Institute of Botany, Guangxi Zhuang Autonomous Region and the Chinese Academy of Sciences, Yanshan, Guilin 541006, China; Macau Centre for Research and Development in Chinese Medicine, The State Key Laboratory of Mechanism and Quality of Chinese Medicine, Institute of Chinese Medical Sciences, University of Macau, Macao SAR 999078, China; College of Pharmacy, Fujian University of Traditional Chinese Medicine, Fuzhou 350122, China; Fujian Provincial Key Laboratory of Haixia Plant Systems Biology, Center for Genomics, College of Life Science, Fujian Agriculture and Forestry University, Fuzhou 350002, China; Fujian Provincial Key Laboratory of Haixia Plant Systems Biology, Center for Genomics, College of Life Science, Fujian Agriculture and Forestry University, Fuzhou 350002, China; Fujian Provincial Key Laboratory of Haixia Plant Systems Biology, Center for Genomics, College of Life Science, Fujian Agriculture and Forestry University, Fuzhou 350002, China; Laboratory of Pharmaceutical Plant Cell Culture Research, School of Biological Engineering, Dalian Polytechnic University, Dalian 116034, China; Fujian Provincial Key Laboratory of Haixia Plant Systems Biology, Center for Genomics, College of Life Science, Fujian Agriculture and Forestry University, Fuzhou 350002, China; Fujian Provincial Key Laboratory of Haixia Plant Systems Biology, Center for Genomics, College of Life Science, Fujian Agriculture and Forestry University, Fuzhou 350002, China

## Abstract

*Clinacanthus nutans* is a traditional medicinal plant widely used in Southeast Asia for treating inflammation, viral infections, and cancer. However, its molecular basis remains poorly understood. In this study, the first chromosome-scale genome of *C. nutans* (731.61 Mbp) was assembled, with 93.76% anchored to 18 pseudochromosomes. Repetitive elements constituted 69.05% of the genome, predominantly long terminal repeat retrotransposons. Phylogenomic and synonymous substitution rate analyses revealed a Lamiales-wide whole-genome duplication event, followed by extensive chromosomal rearrangements. Gene family expansion analysis showed that segmental and dispersed duplications were the primary drivers of enzyme-coding genes (EGs) expansion involved in the flavonoid and triterpenoid pathways. Integrated transcriptomic and metabolomic analyses across five organs revealed distinct organ-specific expression and metabolite profiles. Genes exhibited pronounced differential expression between leaves and roots, with enrichment in flavonoid and triterpenoid biosynthetic pathways, highlighting functional divergence and metabolic specialization. Flavonoids were enriched in aerial tissues, whereas triterpenoids accumulated in roots. Weighted gene co-expression network analysis identified key EGs (e.g. CHS, CHI, OSC) and core transcription factors (e.g. MYB, bHLH, WRKY) potentially involved in organ-specific metabolic regulation. These findings suggest a coordinated transcriptional-metabolic regulatory framework underlying the specialized functions of different tissues. This work provides valuable genomic resources and mechanistic insights into the biosynthesis and regulation of bioactive compounds in *C. nutans*, thereby facilitating future research and molecular breeding of this important ethnomedicinal plant.

## Graphical Abstract

**Graphical Abstract:**
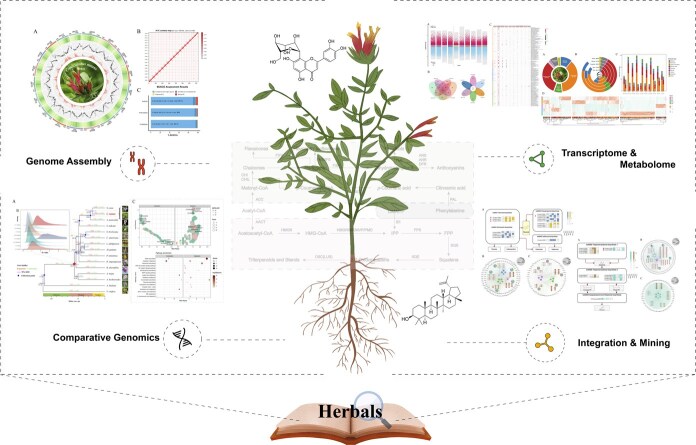

## Introduction

Medicinal plants have been widely utilized in traditional medicine and ethnomedicine worldwide, providing critical contributions to human health over thousands of years. Their therapeutic effects are largely derived from accumulated empirical knowledge and hold irreplaceable significance in many indigenous medical systems [1]. However, despite the remarkable pharmacological activities demonstrated by numerous medicinal plants in both folk usage and clinical practice, the underlying molecular mechanisms and biosynthetic regulatory networks of their active constituents remain largely unexplored [2]. Most previous studies have focused on the isolation and identification of chemical compounds or the evaluation of *in vitro* pharmacological properties [3], while systematic genome-level investigations are still lacking. In particular, research into the biosynthetic pathways of key secondary metabolites, their transcriptional regulatory networks, and patterns of organ-specific accumulation remains limited [4]. The absence of high-quality genomic and transcriptomic data continues to hinder the scientific elucidation of pharmacological efficacy, the development of standardized quality control systems, and the advancement of conservation and molecular breeding efforts.

In recent years, the rapid advancement of high-throughput sequencing technologies, particularly third-generation sequencing platforms such as PacBio HiFi, short-read sequencing technologies such as Illumina, and chromosome conformation capture technologies like Hi-C, has greatly facilitated the construction of high-quality reference genomes in plants, as well as the functional characterization of genes and elucidation of metabolic pathways [5]. These cutting-edge approaches have been widely applied to representative medicinal plant species, including *Perilla frutescens* [6], *Ligusticum chuanxiong* [7], and *Panax ginseng* [8], significantly accelerating the discovery of gene resources and the molecular dissection of bioactive compound biosynthesis. However, many ethnomedicinal plant species have yet to be systematically investigated, with a notable lack of chromosome-scale genome assemblies and comprehensive gene functional annotations [9, 10].

*Clinacanthus nutans*, commonly referred to as ‘ezuihua’ in Chinese, is a member of the Acanthaceae family and represents one of the most widely used ethnomedicinal plants in Southeast Asia. In countries such as Thailand, Malaysia, and Indonesia, its leaves have traditionally been employed in the treatment of inflammatory conditions, herpes simplex virus (HSV) infections, metabolic disorders, and tumor-related symptoms [11]. These traditional uses have been partially supported by pharmacological studies. Phytochemical investigations have revealed that *C. nutans* is rich in flavonoids, triterpenoids, sulfur-containing glycosides, and chlorophyll derivatives [12]. In China, the medicinal applications of *C. nutans* varies across regions, with different parts (leaves, roots, or whole plants) being used for diverse therapeutic purposes [13]. This regional diversity suggests potential organ-specific accumulation of bioactive compounds and pharmacological functions [14]. However, the biosynthetic pathways of these bioactive compounds, the key regulatory genes involved, and their expression patterns under environmental stimuli remain poorly understood [15]. Therefore, the construction of a chromosome-scale reference genome for *C. nutans* using high-throughput sequencing technologies, combined with transcriptome integration and comprehensive gene functional annotation, will provide critical insights into the genetic basis of its medicinal properties. Such genomic resources are essential for elucidating the molecular mechanisms underlying the biosynthesis of key metabolites, and will ultimately support the scientific modernization, sustainable utilization, and conservation of this culturally significant medicinal plant.

In this study, we report a chromosome-scale reference genome assembly of *C. nutans*, constructed using a combination of three sequencing platforms: PacBio high-fidelity (HiFi) single-molecule long-read sequencing, chromosome conformation capture (Hi-C) sequencing, and Illumina short-read sequencing. Based on this high-quality genome, we further resolved the evolutionary position and adaptive characteristics of *C. nutans* within the order Lamiales. We also clarified the organ-specific biosynthetic landscape and bioactive compound diversity in different medicinal organs of *C. nutans*, and systematically analyzed the biosynthetic pathways of key secondary metabolites, including flavonoids, triterpenoids, as well as their organ-specific accumulation patterns. By integrating transcriptomic and metabolomic datasets, we successfully identified core enzyme-encoding genes and transcription factors involved in the biosynthesis of flavonoids and triterpenoids. The genomic resources generated in this study provide a foundational platform for elucidating the biosynthetic basis of bioactive compounds in *C. nutans*, and lay the groundwork for future functional gene studies, germplasm conservation, and molecular breeding efforts.

## Results

### Genome assembly and annotation

**Figure 1:**
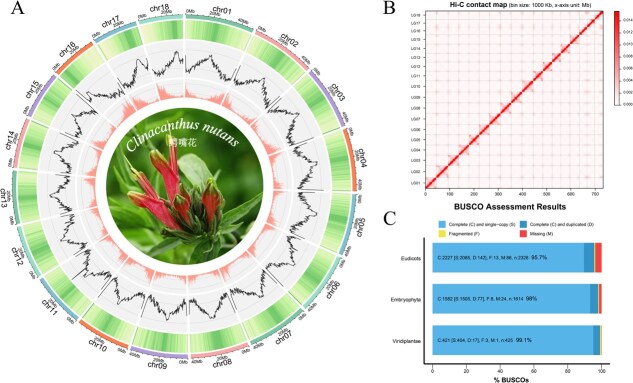
Chromosome-scale genome assembly and quality assessment of *C. nutans.* (A) Circos plot of the *C. nutans* genome at 500 kb resolution. From outer to inner tracks: pseudochromosome IDs (chr01–chr18), GC content, gene density, and distribution of transposable elements (TEs); (B) Hi-C interaction heatmap of the *C. nutans* genome with a bin size of 1000 kb. The interaction intensity is color-coded from the perimeter to the diagonal, representing increasing Hi-C contact frequency; (C) BUSCO completeness assessment of the *C. nutans* genome based on three datasets: embryophyta, eudicots, and viridiplantae.

**Figure 2:**
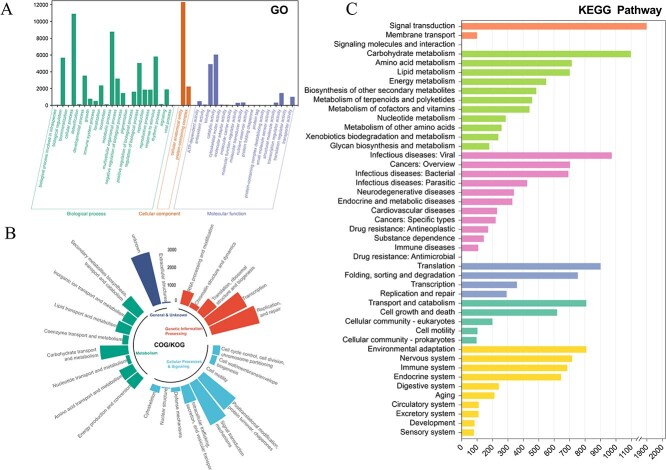
Functional annotation of the *C. nutans* genome: KEGG, GO, and COG classification. (A) Gene Ontology (GO) classification, which includes three major categories: Biological Process (BP), Cellular Component (CC), and Molecular Function (MF); (B) Clusters of Orthologous Groups (COG) classification, where each segment represents a distinct functional category; (C) Kyoto Encyclopedia of Genes and Genomes (KEGG) pathway classification.

We confirmed that *C. nutans* is a diploid species with 36 chromosomes (2*n* = 2*x* = 36) based on metaphase chromosome observations (Fig. S1). The genome size of *C. nutans* was estimated to be approximately 686.86 Mbp based on a K-mer analysis (*K* = 21) using 54.35× Illumina sequencing data, with an estimated heterozygosity of 2.61% (Table S1). This estimate was broadly in line with the flow cytometry result, which indicated a genome size of 0.80–0.84 Gb (Fig. S2). Subsequently, a total of 67.63 Gb of long-read data (~100× coverage) was generated using the PacBio platform. Together with short-read data, a draft assembly of 780.35 Mbp was generated with a contig N50 of 23.68 Mbp (Table S2). Hi-C sequencing (33.02 Gb, ~50× coverage) was employed to build a chromosome-scale assembly. A total of 93.76% of the assembled sequences were anchored to 18 pseudochromosomes (Table S3), resulting in a chromosome-level assembly with a total length of 731.61 Mbp (Fig. 1A and B; Table 1). The completeness of the assembled genome was assessed using BUSCO. The genome captured 99.1%, 98.0%, and 95.7% of conserved genes in the Viridiplantae, Embryophyta, and Eudicots datasets, respectively, indicating high assembly completeness (Fig. 1C). Structural annotation identified 32 943 high-confidence protein-coding genes, with an average gene length of 4332.78 bp and an average of 6.37 exons per gene (Table 1). Additionally, 382 miRNAs, 510 rRNAs, 1068 tRNAs, and 1068 snRNAs were annotated. Functionally, 87.54% of genes were annotated in the eggNOG database, 75.08% in SwissProt, 43.88% in GO, and 26.82% in KEGG. These annotations were primarily associated with DNA replication, signal transduction, transcriptional regulation, and metabolic pathways (Fig. 2, Table S4). Repeat annotation showed that repetitive sequences accounted for 69.05% of the genome. Among them, LTR retrotransposons were the most abundant, comprising 42.96% of the genome, primarily consisting of Gypsy/DIRS1 (33.68%) and Ty1/Copia (7.14%) elements (Table S5).

**Table 1:** Overview of the *C. nutans* genome characteristics.

Genome characteristics	Results
Estimate of genome size (Mb)	698
Total length (bp)	731 613 969
Heterozygous rate (%)	2.58
Contig number	85
Contig N50 size	40 839 156
Largest contig length	48 721 720
GC content (%)	36.05
Number of genes	32 943
Number of transcripts	35 830
Average gene length	4333
Average exon length	289
Average intron length	561
Average exons per gene	6.37
Repetitive sequences and percentage	325 465 719 (44.49%)
miRNAs	382
rRNAs	510
tRNAs	1101
snRNAs	1513

### Comparative analysis of genome evolution

**Figure 3:**
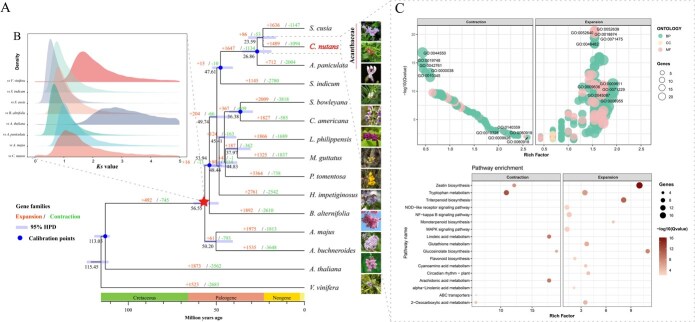
Comparative genomic analyses of *C. nutans* and its closely related species. (A) Phylogenetic tree construction, divergence time estimation, and analysis of orthologous gene cluster expansion and contraction based on single-copy orthologous gene clusters; (B) WGD analysis of *C. nutans* and its closely related species; (C) GO and KEGG enrichment analysis of genes within significantly expanded and contracted orthologous gene clusters in *C. nutans*.

To explore the genomic characteristics and evolutionary relationships of *C. nutans* within the order Lamiales, we performed a comparative genomic analysis using protein-coding genes from *C. nutans* and 14 related species (Table S6). A total of 30 713 genes (93.63% of all protein-coding genes) in *C. nutans* were clustered into 15 278 orthologous gene families. Among the 25 726 total orthogroups identified across all 15 species, 5815 were shared by all species, and 340 represented single-copy orthogroups, serving as reliable markers for phylogenetic inference (Table S7). A phylogenetic tree was constructed based on a supergene concatenated from single-copy orthologous genes using maximum likelihood methods, with five fossil calibrations applied for molecular clock dating (Fig. S3). The resulting tree topology was highly consistent with the APG IV system (Fig. 3A). Within Lamiales, Plantaginaceae was identified as the earliest-diverging lineage, followed by Scrophulariaceae. Bignoniaceae, Paulowniaceae, and Orobanchaceae formed a well-supported clade, where Bignoniaceae was basal, and Paulowniaceae and Orobanchaceae grouped as sister families. Lamiaceae emerged as the most recently diverged lineage in Lamiales. Interestingly, *Callicarpa americana* (formerly classified in Verbenaceae) clustered with *Salvia miltiorrhiza* (Lamiaceae) with strong bootstrap support, in line with recent reclassification of Callicarpa into Lamiaceae [16]. Within Acanthaceae, *Andrographis* was the earliest diverging genus, while *C. nutans* and *S. cusia* formed a sister lineage, indicating their close evolutionary relationship. Divergence time estimation revealed that Lamiales originated from a most recent common ancestor (MRCA) around 56 million years (Mya) ago, shortly after the Cretaceous–Paleogene (K–Pg) boundary and within the Paleocene–Eocene thermal maximum (PETM), a period marked by rapid global warming and ecological turnover [17]. Within Acanthaceae, the divergence between *C. nutans* and *S. cusia* occurred approximately 24.4 Mya, and that between *C. nutans* and *A. paniculata* at ~27 Mya, suggesting a relatively recent diversification within the family.

To further explore genome evolution, whole-genome duplication (WGD) analysis based on Ks distribution revealed a distinct peak among *C. nutans* paralogous genes, suggesting a single WGD event in its history. Comparative Ks analysis between *C. nutans* and *Vitis vinifera* indicated that the WGD occurred after their divergence (Fig. 3B). Additional comparisons with six Lamiales species confirmed that *C. nutans* shares this WGD event with other lineages, rather than undergoing a species-specific duplication. Molecular clock calibration dated this WGD event to approximately 40.2–46.8 Mya, coinciding with the early diversification of Lamiales. These findings suggest that the evolutionary innovations and diversification of *C. nutans*, including its specialized medicinal properties, likely resulted from post-WGD gene retention and functional divergence.

### Comparative analysis of genomic dynamics

Gene family expansion and contraction analysis revealed that 1489 orthogroups were expanded and 1094 orthogroups were contracted in *C. nutans* (Fig. 3A). Among these, 101 gene families (containing 306 protein-coding genes) were significantly expanded, while 14 families (comprising 92 genes) showed significant contraction. GO enrichment analysis of the significantly expanded gene families revealed enrichment in biological processes related to environmental responsiveness and immunity (Fig. 3C, Table S8), such as ‘response to wounding’ (GO:0009611), ‘cellular response to acid chemical’ (GO:0071229). Conversely, genes from significantly contracted families were mainly associated with auxin transport (GO:0060918), auxin import (GO:0060919), and ABC-type transporter activity (GO:0140359). KEGG pathway enrichment further indicated that expanded gene families in *C. nutans* were functionally involved in secondary metabolite biosynthesis (e.g. flavonoids, terpenoids, glucosinolates), plant hormone signal transduction, and stress-related pathways such as the MAPK and NOD-like receptor signaling pathways (Fig. 3D). To gain further insights into the species-specific evolutionary characteristics and evolutionary dynamics of *C. nutans*, we constructed a Venn diagram based on orthologous gene clusters among the three Acanthaceae species: *C. nutans*, *A. paniculata*, and *S. cusia*. A total of 13 073 orthogroups were shared among all three species, while 913 orthogroups were unique to *C. nutans* (Fig. S4A). KEGG enrichment analysis of genes within the *C. nutans* specific orthogroups revealed significant overrepresentation (*Q* < 0.05) in secondary metabolic pathways, such as phenylpropanoid biosynthesis, flavonoid biosynthesis, triterpenoid (steroid) biosynthesis, metabolism of xenobiotics by cytochrome P450, and plant secondary metabolites (Fig. S4B).

**Figure 4:**
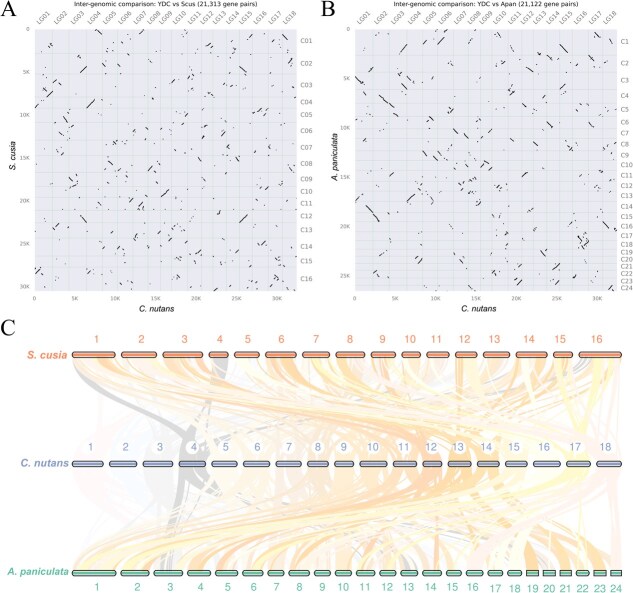
Synteny analysis of the genome of *C. nutans* and its closely related species. (A) Synteny dot plot between *C. nutans* and *S. cusia*; (B) Synteny dot plot between *C. nutans* and *A. paniculata*; (C) Chromosomal synteny analysis among three Acanthaceae species

To further assess chromosomal conservation and structural variation among these Acanthaceae species, genome-wide synteny analyses were performed between *C. nutans* and *A. paniculata* and *S. cusia* (Fig. 4C). Although these species belong to the same family, synteny dot plots revealed fragmented and discontinuous alignments, indicating extensive genome rearrangements (Fig. 4A and B). In total, 638 syntenic blocks comprising 21 313 genes were identified between *C. nutans* and *S. cusia*. Between *C. nutans* and *A. paniculata*, 642 syntenic blocks (covering 21 122 genes) were identified (Table S9). These results suggest a high level of chromosomal structural divergence within Acanthaceae, reflecting substantial genome rearrangements during evolution.

### Transcriptome landscape across different organs in *C. nutans*

**Figure 5:**
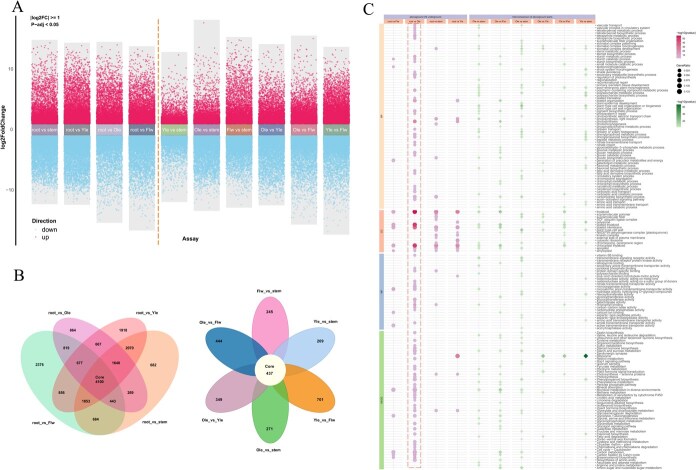
Differential gene expression analysis and functional enrichment results in different organs of *C. nutans.* (A) Volcano plots showing upregulated and downregulated differentially expressed genes (DEGs) across 10 pairwise comparisons between different tissues; (B) Venn diagram (aboveground vs. underground tissues) and petal diagram (comparisons among aboveground tissues) illustrating the overlap of DEGs among different comparisons; (C) dot plot displaying GO and KEGG enrichment analysis results for DEGs across the 10 pairwise comparisons

Transcriptomic sequencing was performed for five tissues of *C. nutans*, including root, stem, young leaf (Yle), mature leaf (Ole), and flower (Flw), generating raw read count matrices and TPM-normalized expression matrices (Table S10). Based on the resulting gene expression profiles, hierarchical clustering and principal component analysis (PCA) were conducted to assess transcriptional variation among tissues. In addition, a Pearson correlation matrix of biological replicates was generated to evaluate sample reproducibility. These analyses showed that biological replicates from the same tissue clustered tightly, indicating high within-group consistency and sample quality (Fig. S5). Transcriptome-wide differences were evident between tissues, particularly between the underground (root) and aboveground (stem, Yle, Ole, Flw) parts. Differential gene expression analysis identified substantial transcriptional divergence across tissues (Fig. 5A, Table S11). A total of 9479 to 13 781 differentially expressed genes (DEGs) were found between the root and each aboveground organ, with the greatest number observed in the comparison between root and young leaf (13 781 DEGs). In contrast, comparisons among aboveground organs yielded fewer DEGs (6856–13 173), highlighting more pronounced functional divergence between root and shoot systems. A total of 4100 DEGs were shared across all root–shoot comparisons, while 437 were shared among aboveground tissues (Fig. 5B).

GO and KEGG enrichment analyses of DEGs revealed functional differentiation consistent with organ-specific roles. Among all pairwise comparisons, the contrast between roots and mature leaves exhibited the largest number of enriched GO terms and KEGG pathways, indicating the most pronounced functional divergence between these two organs (Fig. 5C, Table S12). In root vs. shoot comparisons, DEGs were enriched in photosynthesis (GO:0015979), chloroplast thylakoid function, and primary metabolism in leaves, while roots were associated with secondary metabolism and transport processes. Notably, genes upregulated in mature leaves were enriched in photosynthesis-related pathways and the biosynthesis of flavonoids and phenylpropanoids, which are involved in antioxidative defense. Conversely, root-expressed genes were associated with starch metabolism, carbohydrate transport, and biosynthetic pathways for terpenoids (e.g. sesquiterpenoids, triterpenoids), carotenoids, and vitamin E, which are linked to anti-inflammatory and antimicrobial properties (Fig. S6). These results provide integrated molecular and metabolic evidence for the organ-specific differentiation of medicinal components in *C. nutans*, thereby supporting the rational exploration of organ-targeted utilization strategies.

### Metabolic profile across different organs in *C. nutans*

**Figure 6:**
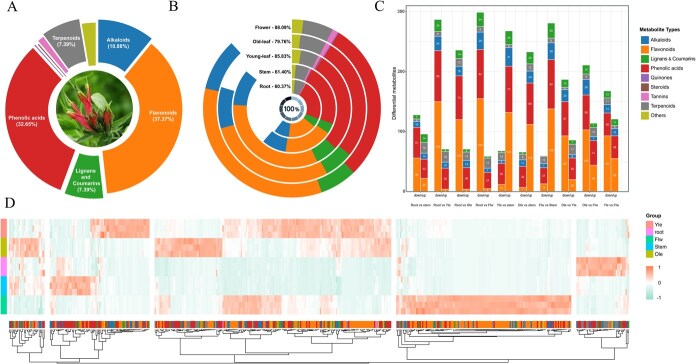
The basic profile of metabolites in *C. nutans.* (A) Pie chart showing the distribution of metabolite classes; (B) bar chart showing the distribution of metabolites in different parts; (C) bar chart of differential metabolite category distribution across different comparison groups; (D) heatmap of metabolomic clustering across different plant parts

To investigate the metabolic landscape of different organs in *C. nutans*, a comprehensive metabolomic analysis was performed using UPLC–MS/MS on the same five tissues used for transcriptomic analysis. A total of 487 metabolites were detected and classified into major categories, with flavonoids (182, 37.37%) and phenolic acids (159, 32.65%) being the most abundant (Fig. 6A, Table S13). Organ-specific distributions were evident, with the root enriched in terpenoids, the flower in phenolic acids and lignans, and the young leaf in flavonoids and alkaloids (Fig. 6B). Differential accumulation analysis across all ten pairwise comparisons revealed 224–358 significantly changed metabolites per group, representing 46.00–73.51% of the total metabolite pool. Terpenoids were predominantly accumulated in roots, while flavonoids and phenolic acids were enriched in aerial parts (Fig. 6C and D). KEGG enrichment analysis showed that these differential metabolites were significantly involved in secondary metabolic pathways such as flavonoid biosynthesis, phenylpropanoid biosynthesis, and terpenoid backbone biosynthesis, as well as core metabolic pathways including glycolysis, amino acid metabolism, and carbon fixation. Notably, flavonoid biosynthesis was among the top enriched pathways across all comparisons, highlighting its crucial role in organ-specific metabolic functions (Fig. S7).

**Figure 7:**
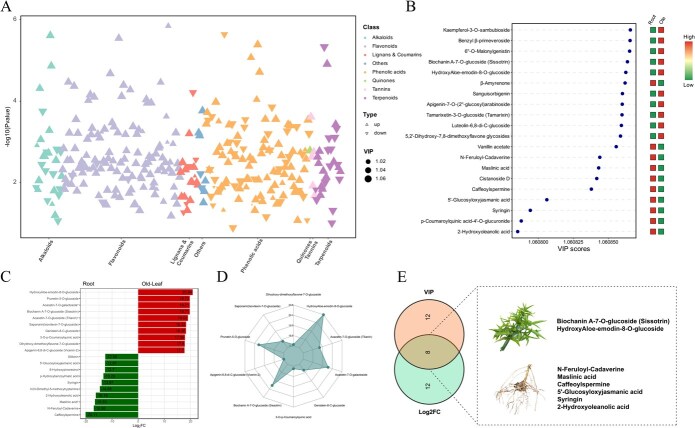
Screening of core differential metabolites between roots and mature leaves. (A) Scatter plot of differential metabolite classification; (B) TOP 20 differential metabolites VIP value plot and heatmap; (C) bar chart of fold change and radar chart of differential metabolites; (D) Venn diagram of TOP 20 differential metabolites based on VIP values and fold change, with a boxed section highlighting characteristic metabolites in the intersection from roots and mature leaves

K-means clustering further categorized 487 differential metabolites into nine clusters based on their accumulation patterns (Fig. S8A, Table S14). Root-enriched clusters (e.g. Cluster 1) were dominated by terpenoids and phenolic acids, while leaf- and flower-enriched clusters (e.g. Clusters 3, 4, 6) featured large numbers of flavonoids, especially flavonols and C-glycosyl flavones. Radar plot and Venn diagram analyses confirmed a clear chemical divergence between underground and aboveground parts (Fig. S8B and C), with 148 metabolites commonly differentially accumulated across all shoot vs. root comparisons, primarily flavonoids [62] and phenolic acids [47]. Further focusing on the comparison between root and mature leaf, 20 core differential metabolites with the highest VIP scores and fold changes were identified (Fig. 7A–D). Flavonoids such as kaempferol, luteolin, and apigenin glycosides were predominantly enriched in leaves, while terpenoids (e.g. oleanolic acid derivatives), alkaloids, and phenolic acids were more abundant in roots (Fig. 7E). The enrichment of antioxidant and UV-protective compounds in leaves, together with the accumulation of anti-inflammatory and antimicrobial constituents in roots, is compatible with the survival strategies of *C. nutans*, reflecting functional differentiation between aboveground and underground organs in response to contrasting environmental pressures. In this context, the observed organ-specific metabolite profiles also show a general correspondence with the differentiated medicinal uses of different plant parts.

### Key modules and regulatory networks related to flavonoid and triterpenoid biosynthesis

**Figure 8:**
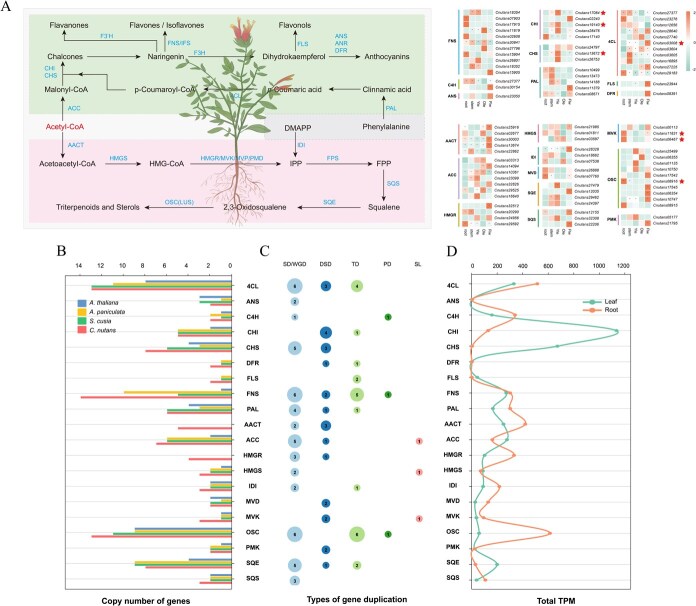
Expression patterns and gene copy numbers of flavonoid and triterpenoid biosynthetic pathways in *C. nutans.* (A) Proposed metabolic pathway and gene expression profiles of flavonoid and triterpenoid biosynthesis in *C. nutans.* EGs expression pattern in the root, stem, young leaf, mature leaf, and flower were simultaneously profiled, with only genes exhibiting TPM > 1 in at least one tissue displayed in the heatmap. Genes marked with a red asterisk are those that overlap with the hub genes identified in the WGCNA analysis; (B) Copy number of EGs in *A. thaliana*, *S. cusia*, *A. paniculata*, and *C. nutans*; (C) Number of genes derived from different types of gene duplication in the genome of *C. nutans*; (D) Comparison of expression levels of EGs between mature leaf and root tissues. Abbreviations: 4CL, 4-coumarate coenzyme A ligase; ANS, anthocyanidin synthase; C4H, cinnamate-4-hydroxylase; CHI, chalcone isomerase; CHS, chalcone synthase; DFR, dihydroflavonol 4-reductase; FLS, flavonol synthase; FNS, flavone synthase; PAL, phenylalanine ammonia-lyase; AACT, acetoacetyl-CoA thiolase; ACC, acetyl-CoA carboxylase; HMGR, 3-hydroxy-3-methylglutaryl-CoA reductase; HMGS, 3-hydroxy-3-methylglutaryl-CoA synthase; IDI, isopentenyl-diphosphate delta-isomerase; MVD, mevalonate-5-pyrophosphate decarboxylase; MVK, mevalonate kinase; OSC, oxidosqualene cyclase; PMK, phosphomevalonate kinase; SQE, squalene epoxidase; SQS, squalene synthase.

To systematically investigate the organ-specific biosynthesis of flavonoids and triterpenoids in *C. nutans*, we first identified 54 and 53 enzyme-encoding genes (EGs) related to flavonoid and triterpenoid biosynthetic pathways, respectively, based on literature mining and KEGG/Pfam database annotations, using both BLAST alignment and HMM domain searches. By integrating transcriptomic expression profiles across five organs, we generated pathway diagrams and gene expression heatmaps for both metabolite classes (Fig. 8A, Table S15), highlighting the spatial distribution of EGs within the biosynthetic pathways. Compared with *Arabidopsis thaliana* and two related species (*A. paniculata, S. cusia*), EGs in *C. nutans* have undergone varying degrees of expansion (Fig. 8B). To further investigate the underlying mechanisms of such expansions, we examined the duplication patterns of genes involved in flavonoid and triterpenoid biosynthetic pathways (Fig. 8C). The expansion of gene families associated with these pathways was primarily attributed to segmental duplications (SD), accounting for 44.44% of flavonoid-related and 52.83% of triterpenoid-related duplicated genes. This was followed by dispersed duplications (DSD) (25.93% and 22.64%, respectively) and tandem duplications (TD) (25.93% and 16.98%, respectively). In contrast, proximal duplications (PD) were relatively rare, contributing only two (3.70%) and one (1.89%) gene pairs to the flavonoid and triterpenoid pathways, respectively.

In the flavonoid biosynthetic pathway, the flavone synthase (FNS) gene family exhibited the most extensive expansion, with a total of 14 duplicated copies, predominantly arising from segmental duplications [6] and tandem duplications [5]. The 4-coumarate: CoA ligase (4CL) gene family included six segmentally duplicated genes, three dispersed copies, and four tandemly duplicated genes localized on chromosome 5. Most were segmentally duplicated, with an additional three copies resulting from dispersed duplication. The chalcone isomerase (CHI) gene family primarily expanded via dispersed duplication, while PAL genes also showed moderate expansion via both segmental and tandem modes. In the triterpenoid pathway, the oxidosqualene cyclase (OSC) gene family underwent remarkable expansion, with six tandem and six segmental duplicates. Notably, the six tandemly duplicated OSC genes were located on chromosomes 1, 3, 5, 6, 10, and 15, respectively. Other key enzymes such as squalene epoxidase (SQE), squalene epoxidase (MVK), and 3-hydroxy-3-methylglutaryl-CoA reductase (HMGR) also exhibited notable expansions, mainly through segmental, dispersed, or tandem duplication events. Several of the duplicated genes exhibited organ-specific expression patterns. For instance, CHI genes showed consistently high expression in mature leaves, whereas OSC genes were predominantly expressed in roots (Fig. 8D). In summary, gene family expansions related to flavonoid and triterpenoid biosynthesis, primarily driven by segmental and dispersed duplications, are widespread in the *C. nutans* genome, collectively shaping its unique genomic architecture and functional landscape. These events likely increased the dosage and expression of rate-limiting enzymes, thereby enhancing metabolic flux and promoting the accumulation of pharmacologically important phenylpropanoid and flavonoid compounds.

**Figure 9:**
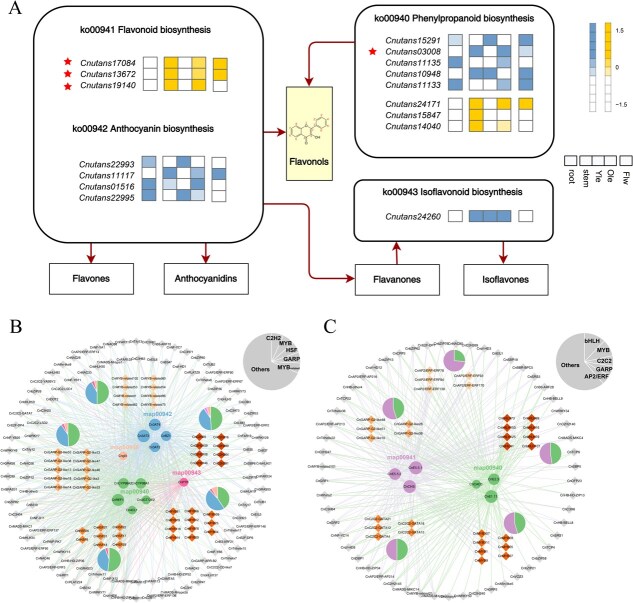
Key EGs and their transcriptional regulatory networks involved in flavonoid biosynthesis. (A) Distribution of key EGs from the blue and yellow modules across flavonoid biosynthetic Pathways. The heatmap color of each gene corresponds to its respective module, and genes marked with red asterisks represent functionally characterized genes in the metabolic pathways; (B) Network Diagram of EGs-TFs Gene Pair in the Blue Module; (C) Network Diagram of EGs-TFs Gene Pair in the Yellow Module. The grey pie chart in the upper right corner represents the proportion of the top five TF families in the network. The circular modules in different colors at the center denote distinct KEGG pathways, while the five diamond-shaped modules around the periphery represent the top five TF families in the network. The colored pie charts above these TF family modules indicate the proportion of metabolic pathways associated with each TF family. The color of each outer circle corresponds to the color of the respective metabolic pathway

To investigate the transcriptional regulatory mechanisms underlying the organ-specific accumulation of flavonoids and triterpenoids in *C. nutans*, weighted gene co-expression network analysis (WGCNA) was conducted by integrating transcriptomic data from five organs with metabolite abundance data for two major classes of secondary metabolites (Tables S9 and S11). Among the eight gene co-expression modules identified for flavonoid biosynthesis-related genes and transcription factors, the blue and yellow modules exhibited strong positive correlations with the accumulation patterns of flavonols and flavones, respectively (Fig. S9). A total of 16 candidate key EGs with high module membership (MM > 0.8) were identified from these two modules. Notably, four genes were functionally assigned to the upstream phenylpropanoid pathway (ko00940), including the 4CL (*Cnutans03008*), which exhibited high transcript abundance specifically in mature leaves, aligning with the organ-specific accumulation of flavonol derivatives. Additionally, two genes in the yellow module, *Cnutans13672* and *Cnutans17084*, were annotated as chalcone synthase (CHS) and CHI, respectively, which catalyze the core reactions of flavonoid skeleton formation. These genes were highly expressed in flowers and mature leaves, consistent with the observed enrichment of flavones in aerial parts (Fig. 9A). TFs-EGs network analysis further revealed MYB, bHLH, and AP2/ERF as core transcription factor families potentially regulating these biosynthetic genes (Fig. 9B and C), suggesting a role for coordinated transcriptional regulation of flavonoid pathways in an organ-specific manner.

**Figure 10:**
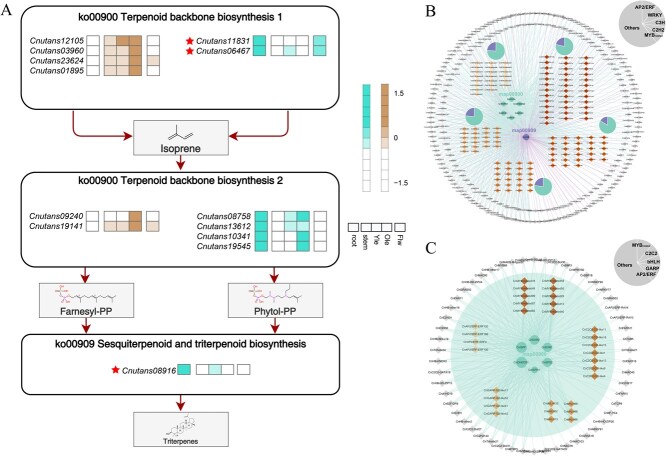
Key EGs and their transcriptional regulatory networks involved in Triterpenoid biosynthesis. (A) Distribution of key EGs from the turquoise and brown modules across triterpenoid biosynthetic pathways. The heatmap color of each gene corresponds to its respective module, and genes marked with red asterisks represent functionally characterized genes in the metabolic pathways; (B) network Diagram of EGs-TFs Gene Pair in the Turquoise Module; (C) network Diagram of EGs-TFs Gene Pair in the Brown Module. The grey pie chart in the upper right corner represents the proportion of the top five transcription factors (TFs) in the network. The circular modules in different colors at the center denote distinct KEGG pathways, while the five diamond-shaped modules around the periphery represent the top five TF families in the network. The colored pie charts above these TF family modules indicate the proportion of metabolic pathways associated with each TF family. The color of each outer circle corresponds to the color of the respective metabolic pathway

For triterpenoid biosynthesis, WGCNA identified eight gene co-expression modules associated with triterpenoid-related EGs and TFs, among which the turquoise and brown modules showed the strongest correlations with the accumulation patterns of triterpenoid compounds. A total of 13 candidate key EGs with high module membership were identified, covering multiple steps of the MVA pathway and downstream triterpene scaffold formation (Fig. S10). Notably, *Cnutans08916*, encoding an OSC, exhibited pronounced root-specific expression and represents a key enzyme responsible for the cyclization of 2,3-oxidosqualene into triterpene skeletons. *Cnutans11831*, annotated as an MVK, plays a critical role in the early steps of the MVA pathway and also showed preferential expression in roots (Fig. 10A). Several other key EGs within these modules, including genes involved in precursor supply and downstream modification steps, displayed similar root-biased expression patterns, collectively supporting an active triterpenoid biosynthetic capacity in underground tissues. Further network analysis highlighted members of the WRKY, C2H2, and AP2/ERF families as potential regulators associated with these key EGs (Fig. 10B and C). These TFs formed tightly connected subnetworks within the turquoise and brown modules, implying their involvement in the coordinated transcriptional regulation of triterpenoid biosynthesis in a root-specific manner.

## Discussion

### Genome assembly, annotation, and comparative genomic analysis

With the rapid advancement and decreasing cost of genome sequencing technologies, high-resolution genomic data have become increasingly available for a wide range of medicinal plant species [18]. To date, chromosome-level genome assemblies have been completed for two major medicinal species in Acanthaceae: *Strobilanthes cusia* and *Andrographis paniculata*. S. *cusia* possesses a relatively large genome of ~865 Mb, with repetitive elements accounting for 79.02% of the genome, mainly due to extensive expansion of LTR retrotransposons [19]. In contrast, *A. paniculata* has a much smaller genome (~269 Mb) with a repeat content of only 57.35%, indicating a more conserved structure and limited transposon activity during its evolution [20]. In this study, we assembled the first chromosome-scale reference genome of *C. nutans*, totaling 731.61 Mb. The repeat content was 69.05%, dominated by LTR retrotransposons (~43%), especially Gypsy and Copia elements. This level of repeat expansion places *C. nutans* between *S. cusia* and *A. paniculata*, suggesting a moderate degree of genome inflation during its evolutionary history. Expansion of repetitive sequences, particularly LTR retrotransposons, is a major contributor to genome size variation in plants. The intermediate genome size and repeat proportion in *C. nutans* strongly suggest that its genome expansion was primarily driven by transposon activity. Such structural changes not only affect genome architecture and stability but may also influence gene regulation and secondary metabolism through the insertion of regulatory elements.

Previous studies have demonstrated that a WGD event occurred prior to the divergence of families within the order Lamiales, which played a pivotal role in the establishment and rapid radiation of this lineage [21]. Our Ks analysis further confirmed the presence. The neo-polyploid genomes are prone to structural reshuffling, including chromosomal breakage and rearrangement, gene loss, copy number variation, and changes in chromosome number, processes that collectively accelerate genomic evolution [22]. Acanthaceae, in particular, exhibits pronounced karyotypic diversity and structural genome plasticity, traits that are believed to contribute to its adaptive diversification [23]. Our comparative synteny analysis revealed unexpectedly low collinearity between *C. nutans* and its congeneric relatives (*A. paniculata* and *S. cusia*), with only a small proportion of syntenic blocks aligned to homologous chromosomes. This pattern suggests extensive chromosomal rearrangements have occurred during the evolutionary history of this family. Taken together, the genomic landscape (repeat composition and synteny structure) of *C. nutans* highlight its unique evolutionary trajectory within Acanthaceae and provides a critical genomic foundation for its medicinal functions.

Numerous studies have demonstrated that different modes of gene duplication contribute differentially to the formation of regulatory networks, the acquisition of novel functions, and adaptive evolution in plants [24, 25]. In *C. nutans*, our results revealed that DSD and WGD/SD are the predominant duplication types associated with the expansion of key EGs in the flavonoid and triterpenoid biosynthetic pathways (Fig. 8C). Segmental duplication events, often remnants of ancient WGD processes, are known to preserve syntenic gene pairs and preferentially retain dosage-sensitive genes, such as transcription factors and core enzymes in signaling and metabolic pathways [26]. This duplication mode plays a critical role in the systemic construction and stabilization of complex regulatory networks and provides a foundational framework for the evolution of elaborate plant traits. In contrast, dispersed duplications exhibit greater structural flexibility and functional variability. These gene copies are scattered across the genome and are more susceptible to diversifying selection, which facilitates neofunctionalization and the emergence of lineage-specific metabolic functions [27, 28]. The coexistence of these two major duplication types in *C. nutans* suggests a complementary model of gene family evolution, in which WGD/SD events contribute to a conserved regulatory backbone, whereas DSD promotes local innovation and functional divergence, and may contribute to shaping the evolutionary landscape of genes associated with secondary metabolism.

### Transcriptomic and metabolomic insights into organ-specific biosynthesis and bioactive diversity

One of the main objectives of this study was to elucidate the ‘therapeutic wisdom’ of *C. nutans*, by characterizing the organ-specific distribution patterns and variability of its bioactive constituents. Transcriptomic comparisons among five organs revealed pronounced transcriptional divergence between the underground (root) and aerial (leaf, stem, flower) parts. A total of 4100 DEGs were shared among root–shoot comparisons, indicating extensive spatial regulation of gene expression. Functional enrichment analyses showed that genes preferentially expressed in roots were mainly involved in starch and carbohydrate metabolism, amino acid catabolism, and triterpenoid biosynthesis. These pathways have been previously associated in the literature with biological activities such as anti-inflammatory and antimicrobial effects, as well as vascular regulation [29–31]. In contrast, mature leaves showed enhanced expression of genes related in photosynthesis, pigment production, and flavonoid biosynthesis, a class of compounds that has been widely reported to possess antioxidant and antiviral activities [32, 33]. These expression patterns reflect a clear division of physiological functions between the underground and aerial parts and provide molecular evidence supporting ethnomedical practices that differentiate therapeutic applications by tissue.

Metabolomic profiling further supported this organ-specific biochemical differentiation. A total of 182 flavonoids and 36 triterpenoids were identified, displaying distinct organ-enrichment patterns. Flavonoids such as kaempferol, luteolin, and apigenin derivatives were predominantly accumulated in leaf and flower tissues, consistent with their photosynthetic roles and light exposure, as well as with previous reports on flavonoid localization and function [34]. By contrast, roots were enriched in triterpenoids and phenolic acids (e.g. maslinic acid, 2-hydroxyoleanolic acid, syringin), which have been reported to be involved in stress responses and immune modulation [35]. K-means clustering analysis revealed that roots and aerial tissues exhibited distinct metabolic signatures, with roots forming a chemotype dominated by terpenoids and phenolic acids. These metabolite distributions correspond well with the organ-specific expression of biosynthetic genes and are consistent with traditional uses: root for activating blood and relieving swelling, and leaves for antiviral and detoxifying purposes. Overall, our findings reveal a coordinated transcriptional-metabolic framework framework that governs the medicinal-use differentiation of organs in *C. nutans.* While direct pharmacological validation was beyond the scope of this study, these integrative multiomics patterns provide a biologically plausible molecular basis for interpreting traditional, organ-specific medicinal applications and offer a foundation for future functional and bioassay-based investigations.

### Integrated omics analysis reveals key EGs and regulatory modules

In *C. nutans*, the pronounced spatial separation of flavonoid and triterpenoid accumulation between aerial and underground parts reflects functional organ specialization and environmental adaptation. Through WGCNA, we systematically identified modules associated with flavonoid and triterpenoid biosynthesis and pinpointed key EGs and core TFs involved in these pathways. For flavonoids, the blue and yellow modules were significantly correlated with flavonol and flavone subclasses, respectively. Within these modules, key EGs such as CHS (*Cnutans13672*), CHI (*Cnutans17084*), and 4CL (*Cnutans03008*) were highly expressed in leaves and flowers, consistent with the organ-specific accumulation of flavonoids. These enzymes catalyze early steps of the phenylpropanoid and flavonoid pathways [36], and their co-expression with MYB, bHLH, and C2H2 family TFs suggests a conserved regulatory mechanism. Similar TFs have been reported to play central roles in flavonoid regulation in other plants, including *Arabidopsis* [37], *Camellia* [38], and *Salvia* [39]. Triterpenoid biosynthesis was predominantly associated with the turquoise and brown modules, which showed strong correlation with root-enriched metabolites. Genes such as MVK (*Cnutans11831*) and OSC (*Cnutans08916*) were specifically expressed in roots and are known to catalyze early steps and skeleton formation in the mevalonate pathway [40]. These were co-expressed with WRKY, AP2/ERF, and MYB-related TFs, suggesting an organ-specific transcriptional network regulating triterpenoid biosynthesis in roots. The observed spatial correspondence between gene expression patterns and metabolite accumulation offers important clues to the metabolic partitioning of flavonoid and triterpenoid biosynthesis in *C. nutans*. However, it should be emphasized that the regulatory relationships proposed here are inferred from co-expression analyses and therefore do not represent direct functional validation. Accordingly, the identified key EGs and core TFs should be regarded as candidate regulators, and their putative roles are best interpreted as testable hypotheses rather than confirmed mechanisms. Future functional studies, including gene knockout or overexpression approaches and targeted metabolite assays, will be essential to validate these regulatory relationships, refine the proposed regulatory framework, and ultimately establish their roles in controlling flavonoid and triterpenoid biosynthesis in *C. nutans*.

## Conclusion

This study reports a high-quality chromosome-scale genome assembly of *C. nutans* and integrates comparative genomics, transcriptomics, and metabolomics analyses to elucidate the molecular mechanisms underlying its organ-specific accumulation of secondary metabolites. Ks distribution analyses revealed a Lamiales-wide WGD event, providing an evolutionary basis for gene expansion and functional diversification in *C. nutans*. Transcriptomic and metabolomic profiling showed that flavonoids were predominantly accumulated in aerial parts, whereas triterpenoids enriched in underground tissues, both under tight regulation by organ-specific gene expression. WGCNA further identified key EGs and core TFs (e.g. CHS, CHI, OSC, and members of the MYB, bHLH, and WRKY families) potentially involved in the biosynthesis and spatial regulation of secondary metabolites. Collectively, this work establishes a foundation for elucidating the biosynthetic mechanisms of medicinal compounds in *C. nutans* and provides valuable genomic resources to support future functional studies, metabolic engineering, and molecular breeding of this ethnomedicinal plant. More broadly, the integrative multiomics strategy presented here offers a generalizable framework for translating ethnomedicinal knowledge into molecular and genomic insights, which can be readily extended to other medicinal plants with organ-specific therapeutic applications.

## Materials and methods

### Sampling and sequencing of plant materials

A mature *C. nutans* plant cultivated at the Fujian Agriculture and Forestry University in Fujian Province, China, was selected for sequencing. Approximately, 10 g of fresh leaves were collected and immediately frozen in liquid nitrogen. High-quality genomic DNA was extracted using the cetyltrimethylammonium bromide method. For PacBio single-molecule real-time (SMRT) sequencing, at least 10 μg of sheared DNA was used to construct a 20-kb SMRTbell library, which was then sequenced on the PacBio Sequel II platform following the manufacturer’s protocol. For Hi-C sequencing, over 2 g of fresh young leaves were fixed in 1% formaldehyde to crosslink chromatin interactions. Hi-C libraries were constructed according to standard protocols, including chromatin extraction and digestion, DNA ligation, purification, and fragmentation (~350 bp), and sequenced on the Illumina HiSeq 2500 platform. To assist gene annotation, RNA was extracted from root, stem, young leaf, mature leaf, and flower tissues from the same plant using the TRIzol reagent (Invitrogen, USA). RNA integrity was assessed using the Agilent 2100 Bioanalyzer. Libraries were prepared with the NEBNext Ultra RNA Library Prep Kit (NEB, USA) and sequenced using the Illumina HiSeq 2500 platform. In addition, the same tissues were processed for LC–MS analysis for metabolomic profiling.

### Genome assembly and gene annotation

The *C. nutans* genome was assembled at the contig level using Hifiasm [41]. Multiple rounds of assembly were conducted to optimize genome structure according to its complexity and data characteristics. Chromosome-level scaffolding was performed using HapHiC [42], followed by visual assessment. Genome assembly quality was evaluated with BUSCO [43]. QUAST was used to calculate N50, genome size, and GC content [44]. Gene structure annotation was performed using a combination of *ab initio* and homology-based methods. Augustus, SNAP, and GeneWise were used for gene prediction [45, 46], guided by reference species including *Lycopersicon esculentum*, *S. miltiorrhiza*, *S. bowleyana*, *Strobilanthes cusia*, and *A. thaliana*. Tandem repeats and transposable elements were annotated using RepeatMasker [47] and RepeatModeler [48]. Functional annotation of predicted proteins was conducted using eggNOG-mapper [49], based on COG, KOG, KEGG, and GO databases to assign putative biological functions. The resulting dataset provided a comprehensive functional profile of the *C. nutans* genome.

### Comparative genomic analysis of *C. nutans* and its related species

Genome data from 14 published chromosome-level plant genomes related to *C. nutans* were selected for comparative genomic analysis (Table S6). These included 12 Lamiales species: *Antirrhinum majus* [50] (Plantaginaceae), *Adenosma buchneroides* [51] (Plantaginaceae), *Buddleja alternifolia* [52] (Scrophulariaceae), *Sesamum indicum* [53] (Pedaliaceae), *Andrographis paniculata* [20] and *Strobilanthes cusia* [19] (Acanthaceae), *Handroanthus impetiginosus* [54] (Bignoniaceae), *Callicarpa americana* [55] (Lamiaceae), *S. bowleyana* [56] (Lamiaceae), *Mimulus guttatus* [57] (Phrymaceae), *Paulownia tomentosa* [58] (Paulowniaceae), and *Lindenbergia philippensis* [59] (Orobanchaceae). Additionally, *A. thaliana* (Brassicaceae) and *V. vinifera* (Vitaceae) were included as outgroups.

Gene annotation files and coding sequences for all species were standardized prior to analysis. Orthologous gene families across the 15 species were identified using DIAMOND [60] and OrthoFinder [61]. Single-copy orthologues were aligned with MAFFT and concatenated to generate a supergene [62]. Phylogenetic trees were inferred using IQ-TREE [63]. Divergence times were retrieved from the TimeTree database (http://www.timetree.org) and used to calibrate the tree using fossil points. Fourfold degenerate sites (4DTv) were extracted using Perl scripts, and divergence time estimation was performed using MCMCTree [64]. Gene family expansion and contraction were assessed using CAFÉon the time-calibrated phylogenetic tree with parameters -p 0.05 and lambda -s [65]. To investigate the WGD history of *C. nutans*, the dmd module of the wgd toolkit was used to identify syntenic orthologues. Ks values were calculated using the ksd module based on MCL-clustered gene families to plot Ks distribution curves and infer WGD events and divergence timing [66]. Synteny analysis was also performed for *C. nutans*, *S. cusia*, and *A. paniculata*. JCVI (jcvi.compara.catalog) was used to detect collinear blocks within and between genomes. Synteny dot plots and karyotype-level comparative maps were visualized using JCVI tools [67].

### Transcriptome and metabolome analysis

Fresh tissues from five different organs (root, stem, young leaf, mature leaf, and flower) of the same *C. nutans* plant used for genome sequencing were collected and immediately frozen in liquid nitrogen. Total RNA was extracted using the TRIzol reagent (Invitrogen, USA) following the manufacturer’s protocol. RNA integrity was assessed using the Agilent 2100 Bioanalyzer. Libraries were prepared using the NEBNext Ultra RNA Library Prep Kit (NEB, USA) and sequenced on an Illumina HiSeq 2500 platform to generate 150 bp paired-end reads. After quality control using Trim_galore, clean reads were mapped to the assembled genome using HISAT2 [68]. Gene-level read counts were quantified with Subread-featureCounts under default settings. DEGs were identified using edgeR with thresholds of false discovery rate ≤ 0.01 and |log_2_ fold change| ≥ 2 [69]. Gene expression levels were normalized and reported as transcripts per million (TPM).

### Metabolite extraction and UPLC-MS/MS profiling

The same five tissue types were used for metabolomic profiling. Approximately, 100 mg of each tissue was weighed and ground into fine powder under liquid nitrogen. Metabolites were extracted with 70% aqueous methanol overnight at 4°C, followed by centrifugation at 10 000 × g for 10 minutes. The supernatants were filtered through a 0.22-μm membrane and subjected to UPLC-MS/MS analysis using a Waters ACQUITY UPLC HSS T3 C18 column (1.8 μm, 2.1 mm × 100 mm). Metabolite identification and quantification were performed using a self-built database (MetWare, Wuhan, China) and the MWDB database. Data were normalized and subjected to multivariate statistical analyses, including PCA and partial least squares–discriminant analysis (PLS-DA), following the standard MetWare metabolomics workflow. Differential metabolites were identified based on variable importance in projection (VIP > 1) derived from PLS-DA [70], together with fold change (≥2 or ≤0.5).

### Identification of biosynthetic genes and co-expression network construction

To explore genes involved in secondary metabolite biosynthesis, we curated EGs lists based on literature and KEGG pathway annotations. *A. thaliana* orthologues were retrieved from TAIR (www.arabidopsis.org/) and used as seed sequences to construct a BLAST database. Hidden Markov Model (HMM) profiles of relevant domains were downloaded from the Pfam database (http://pfam.xfam.org/). Candidate genes were screened using both BLAST and HMM methods. Functional domains were confirmed using CDD-search and SMART [71, 72], and conserved motifs were assessed using MEME [73]. Genes showing conserved domains and motifs similar to those in *A. thaliana* were selected as biosynthetic pathway candidates (Table S15).

Following the method of Yang et al. [74], we selected EGs and TFs from KEGG pathways associated with flavonoid (ko00940, ko00941, ko00942, ko00943, ko00944, ko00946) and triterpenoid (ko00900, ko00909) biosynthesis. TFs were annotated using iTAK [75]. Expression data of EGs and TFs from transcriptomes were combined with matched metabolite profiles for WGCNA. Genes with TPM < 1 in more than 70% of samples were removed. WGCNA was conducted with parameters: power = 14, R [2] = 0.8, module size = 40, merge threshold = 0.3. Pearson correlation (|r| > 0.8) was used to associate gene modules with metabolite abundance. Key EGs were defined by module membership (MM > 0.8) and edge weight (EW > 0.15). Core TFs were selected based on their co-expression with key EGs. Co-expression networks were visualized using Cytoscape [76].

## Supplementary Material

Web_Material_uhag037

## Data Availability

The genome sequences and raw RNA-seq data described in this study have been deposited in CNGBdb under the accession number CNP0008755. The metabolomic datasets and related project reports are available from the corresponding author upon reasonable request.
